# Physical activity modifies the association between atherogenic index of plasma and prediabetes and diabetes: A cross‐sectional analysis

**DOI:** 10.1111/1753-0407.70006

**Published:** 2024-10-13

**Authors:** Shenglan Yang, Xinyu Gou, Hui Dong, Limei Chen, Yiyan Wang, Jing Wu

**Affiliations:** ^1^ School of Nursing, Shanghai University of Traditional Chinese Medicine Shanghai China

**Keywords:** atherogenic index of plasma, physical activity, prediabetes and diabetes, young and middle‐aged populations

## Abstract

**Background:**

Although research has explored the association between atherogenic index of plasma (AIP) and prediabetes and diabetes, there is still not sufficient available evidence the role of physical activity (PA) in this relationship. Our purpose is to examine the complex connections between AIP, PA, and prediabetes and diabetes in a young and middle‐aged population.

**Methods:**

This study included 2220 individuals from the general population, aged 20–60 years. AIP was calculated from the logarithm of the triglyceride (TG) to high‐density lipoprotein cholesterol (HDL‐C) ratio. PA was assessed depending to the American Heart Association (AHA) criteria and categorized into medium‐high and low PA levels. We used binary logistic regression to explore associations and subsequently performed sensitivity and subgroup analyses.

**Results:**

The 2220 participants had a mean age of 38 years, with a mean AIP of −0.1185, and a prediabetes and diabetes prevalence of 7.2%. After adjusting for auxiliary variables, AIP was positively correlated with prediabetes and diabetes (odds ratio [OR]: 3.447, 95% confidence interval [CI]: 1.829–6.497). In the low PA population, the prevalence of prediabetes and diabetes raised significantly with higher AIP (OR: 3.678, 95% CI: 1.819–7.434). This association was not meaningful in the medium to high PA population (OR: 1.925, 95% CI: 0.411–9.007). Joint and sensitivity analyze results also showed agreement. Restricted cubic spline identified a linear relationship between AIP and the prevalence of prediabetes and diabetes. Notably, the prevalence significantly increases when AIP values exceed −0.16 (*p* for linearity <0.05). The findings revealed heterogeneity across subgroups stratified by sex and age.

**Conclusions:**

PA may modify the link as regards AIP with prediabetes and diabetes in young and middle‐aged populations. Adherence to PA prevents the adverse effects of abnormal glucose metabolism caused by dyslipidemia, particularly in women.

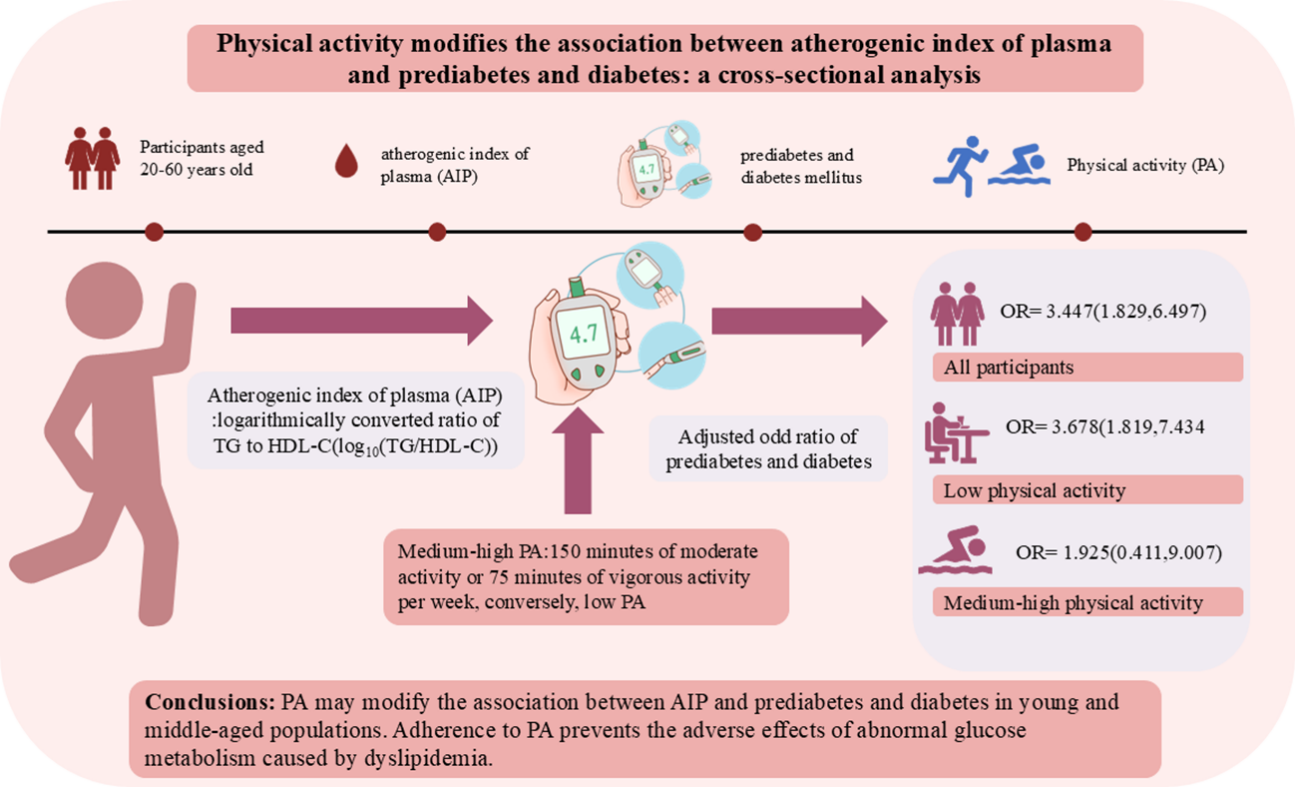

## Graphical abstract

Highlights

- After adjusting for confounders, AIP was positively correlated with prediabetes and diabetes, showing a greater effect in the low PA group and a nonsignificant relationship in the high PA group.
- Restricted cubic spline showed that in midlife populations, AIP was directly associated with prediabetes and diabetes prevalence when AIP > −0.16, and the reverse was not significant.
- PA can modify the link as regards AIP with prediabetes and diabetes, especially in females.

## 1 INTRODUCTION

The global incidence of diabetes is increasing due to longer life expectancy and lifestyle changes, including poor diet and physical inactivity. The International Diabetes Federation (IDF)^1^ predicts that the amount of patients with diabetes will reach 783 million by 2045, significantly increasing the global healthcare burden. Currently, approximately 403 million people aged 20–64 worldwide have diabetes.^2^ Among those aged 20–40, the prevalence is nearly 20%. Additionally, 32.6% of diabetes‐related deaths occur in individuals under 60, highlighting the significant impact of diabetes on younger populations.^2^, ^3^ Notably, a segment of the young and middle‐aged population is in the prediabetes stage, characterized by insulin resistance (IR) and abnormal glucose tolerance.^3^, ^4^ Therefore, preventing and treating both diabetes and prediabetes are crucial to reducing the incidence of diabetes‐related complications and cardiovascular diseases.

Diabetes is one of the major reasons for disability and death.^5^ Younger diabetes people experience more severe metabolic disturbances at onset compared with elderly patients, resulting in a greater risk of developing complications and quicker progression to adverse cardiometabolic profiles.^6^, ^7^ It is well acknowledged that diabetes elevates the risk of atherosclerotic cardiovascular disease (ASCVD).^8^ Studies have shown that patients with atherosclerosis and type 2 diabetes exhibit higher levels of systemic inflammatory markers.^9^ For example, hyperglycemia is associated with the increased production of advanced glycosylation end products (AGEs), which can lead to endothelial dysfunction and plaque formation. Additionally, the lipid profile of diabetic patients typically shows elevated levels of TG and LDL‐C, along with reduced levels of HDL‐C.^9^, ^10^ The relationship between glucose metabolism and lipid metabolism has been controversial for a long time. Recent studies have found that a high TG to HDL‐C ratio is accompanied by an increased risk of new‐onset diabetes.^11^ The AIP, computed from the log‐transformed ratio of TG to HDL‐C, has recently emerged as a superior lipid index.^12^ Increasing studies show that AIP is intimately associated with diabetes and prediabetes and can predict diabetes. Higher AIP probably decreases the amount and activity of insulin receptors on fat cells or causes a drop in insulin secretion and sensitivity, potentially causing diabetes.^13^, ^14^ Data from a study of US adults indicated that more AIP is significantly correlated with more prevalence of prediabetes and diabetes.^15^ It is also valuable for mirroring IR, which is strongly connected to glucose metabolism dysfunction.^16^

PA is frequently recommended for diabetes prevention and control.^17^ PA can increase insulin sensitivity and reduce obesity, improving glucose uptake and controlling blood sugar for diabetes prevention and treatment.^18^, ^19^ A large cohort study showed that a certain intensity of PA is relationship with a lower risk of diabetes.^20^ The American Heart Association (AHA) has identified PA as one of eight modifiable factors to prevent and delay cardiovascular diseases.^21^ However, few researches have explored the complex associated between AIP and PA in relation to diabetes and prediabetes.

There is a positive correlation between PA and diabetes and prediabetes, whereas an inverse relationship exists between AIP and diabetes and prediabetes. Therefore, it is reasonable to assume that PA may attenuate the association between AIP and diabetes and prediabetes, particularly as higher intensity PA mitigates the impact of AIP on the development of prediabetes and diabetes. Our study aims to test this hypothesis in the young and middle‐aged population and to search for the relationships between PA, AIP, diabetes, and prediabetes.

## 2 METHODS

### 2.1 Data source and participants

This ongoing prospective cohort study examines the impact of demographic factors, dietary behavior, blood biochemical parameters, psychological status, and metabolic disease on the development of cardiovascular disease in midlife subjects. Data for this study were collected from July 2022 to May 2023. Incorporation criteria were (1) aged 18–60 years; (2) employed in enterprises or public institutions; (3) capable of normal communication and voluntary signing of informed consent; and (4) complete questionnaires, laboratory tests, and physical examinations. Exclusion criteria included (1) patients with tumors or other chronic wasting diseases; and (2) pregnant women. Trained researchers conducted the study, providing necessary explanations and instructions. A total of 2220 subjects were enrolled in the research. Written informed agreement was received from all involved persons and reviewed and approved by the Medical Ethics Committee of Shanghai Zhoupu Hospital (Project No. 2022‐C‐068‐E01).

### 2.2 Measurement of important variables

AIP is calculated based on the ratio of TG (mmol/L) to HDL‐C (mmol/L), expressed as a logarithmic number on a 10‐point scale: log10 [TG/HDL‐C].^12^ In this study, subjects will be categorized into four groups depending on their AIP quartile levels: quartile 1 (Q1) with AIP < −0.3398, quartile 2 (Q2) with AIP ranging from −0.3398 to −0.1610, quartile 3 (Q3) with AIP ranging from −0.1610 to 0.0613, and quartile 4 (Q4) with AIP > 0.0613.

PA was measured based on patient self‐reports from questionnaires. Researchers assessed PA using the AHA “Life's Essential 8” criteria. We categorized participants as engaging in moderate PA if they engaged in more than 150 min per week of activities, such as water aerobics, social dancing, or gardening, and as engaging in vigorous PA if they engaged in more than 75 min per week of activities, such as running, swimming laps, or jumping rope. If these criteria were not met, the activity level was classified as low.^21^

The outcome variables were prediabetes and diabetes. From the World Health Organization (WHO) and Chinese Diabetes Guidelines,^17^, ^22^ diabetes mellitus or prediabetes is diagnosed if the patient meets one of the following three conditions: FBG level ≥6.1 mmol/L, glycosylated hemoglobin level ≥6.0%, or in an oral glucose tolerance test (OGTT), a blood glucose level ≥7.8 mmol/L within 2 h after taking 75 g of glucose orally. Prediabetes is associated with an elevated risk of progressing to diabetes and its associated complications. The pathogenesis of both conditions is closely linked to IR,^23^, ^24^ Thus, in this study, the combination of prediabetes and diabetes was treated as a single outcome event.^15^

### 2.3 Covariates

We collected socio‐demographic characteristics and blood biochemical indices of the participants through questionnaires and health examinations. Covariates included gender (male or female), age (years), body mass index (BMI) (kg/m^2^), total cholesterol (TC, mmol/L), low‐density lipoprotein cholesterol (LDL‐C, mmol/L), smoking status (yes or no), drinking status (yes or no), marital status (unmarried or married), and education level (below undergraduate or undergraduate and above).

### 2.4 Statistical analysis

We summarized the general characteristics of the study population using descriptive statistics. We used logistic regression to evaluate the relations with AIP (as quartiles or continuous variables) and prediabetes and diabetes, expressed as odds ratio (OR) and 95% confidence interval (CI). We stratified our analyses by low PA and medium to high PA. The aim was to determine if these associations differed by PA level. We also calculated the *p* for trend and plotted the restricted cubic spline (RCS) to assess the results of AIP as a continuous variable and its nonlinear relationship. Based on the critical values from the RCS analysis, AIP was classified as a dichotomous variable and analyzed jointly with PA. The respondents of the study were classified into four teams: low AIP and medium‐high PA (AIP−/PA−), low AIP and low PA (AIP−/PA+), high AIP and medium‐high PA (AIP+/PA−), and high AIP and low PA (AIP+/PA+) to observe the relationship with prediabetes and diabetes. We adjusted three logistic models for different covariates to evaluate the relationship of AIP with prediabetes and diabetes mellitus. The three models are (1) model 1, not adjusted for any covariates; (2) model 2, adjusted for gender and age; (3) model 3, adjusted for gender, age, BMI, TC, LDL‐C, smoking, drinking, marital status, and education.

Additionally, we performed sensitivity and subgroup analyses. Sensitivity analyses involved linear regression of AIP (as a categorical or continuous variable) against fasting blood glucose (FBG) values. Subgroup analyses were stratified by gender and age, including AIP as both a categorical and continuous variable. All three models were adjusted accordingly.

The statistical analysis software SPSS27.0 and RStudio were used for this study. *p* < 0.05 was considered statistically meaningful.

## 3 RESULTS

### 3.1 General characteristics of the study population

The common characteristics of the research subjects are listed in Table 1. The average age is 38 years, the average BMI is 23.13 kg/m^2^, 77.0% are female, 80.1% are married, 72.3% have a bachelor's degree or higher, and 73.5% have a monthly income of less than $8000. Only about 9.1% of the participants smoked and drank alcohol. The prevalence of prediabetes and diabetes was 7.2%, and only 18.5% of participants achieved the AHA's recommended level of PA.

**TABLE 1:** General characteristics of the population.

Variables	Total sample (*n* = 2220)	Q1 <−0.3398	Q2 −0.3398 to −0.1610	Q3 −0.1610 to −0.0613	Q4 >0.0613	*p* value
Age (years)	38.45 ± 8.06	37.18 ± 7.57	38.06 ± 8.27	38.67 ± 8.28	39.90 ± 7.87	<0.001
Gender						<0.001
Male	510 (23.0)	34 (6.1)	73 (13.1)	138 (24.9)	265 (47.7)	
Female	1710 (77.0)	520 (93.9)	483 (86.9)	417 (75.1)	290 (52.3)	
BMI (kg/m^2^)	23.13 ± 3.46	21.28 ± 2.35	22.33 ± 3.00	23.44 ± 3.28	25.49 ± 3.60	<0.001
Marital status						0.033
Unmarried	442 (19.9)	120 (21.7)	128 (23.0)	100 (18.0)	94 (16.9)	
Married	1778 (80.1)	434 (78.3)	428 (77.0)	455 (82.0)	461 (83.1)	
Education						0.065
Under undergraduate	615 (27.7)	132 (23.8)	151 (27.2)	162 (29.2)	170 (30.6)	
Undergraduate and above	1605 (72.3)	422 (76.2)	405 (72.8)	393 (70.8)	385 (69.4)	
Monthly income						0.788
≤8000 yuan	1632 (73.5)	411 (74.2)	413 (74.3)	409 (73.7)	399 (71.9)	
>8000 yuan	588 (26.5)	143 (25.8)	143 (25.7)	146 (26.3)	156 (28.1)	
Smoking						<0.001
No	2017 (90.9)	542 (97.8)	533 (95.9)	502 (90.5)	440 (79.3)	
Yes	203 (9.1)	12 (2.2)	23 (4.1)	53 (9.5)	115 (20.7)	
Drinking						<0.001
No	2018 (90.9)	536 (96.8)	526 (94.6)	512 (92.2)	444 (80.0)	
Yes	202 (9.1)	18 (3.2)	30 (5.4)	43 (7.8)	111 (20.0)	
Physical activity (PA)						0.021
Low PA	1810 (81.5)	442 (79.8)	445 (80.0)	451 (81.3)	472 (85.0)	
Medium‐high PA	410 (18.5)	112 (20.2)	111 (20.0)	104 (18.7)	83 (15.0)	
Glucose (mmol/L)	5.39 ± 0.84	5.16 ± 0.44	5.26 ± 0.47	5.41 ± 0.66	5.73 ± 1.33	<0.001
TC (mmol/L)	5.02 ± 0.86	4.85 ± 0.82	4.89 ± 0.77	5.05 ± 0.86	5.29 ± 0.96	<0.001
TG (mmol/L)	0.97 (0.72,1.47)	0.58 (0.50,0.66)	0.84 (0.75,0.92)	1.17 (1.03,1.32)	2.03 (1.64,2.66)	0.001
HDL‐C (mmol/L)	1.42 ± 0.36	1.72 ± 0.25	1.50 ± 0.21	1.35 ± 0.21	1.13 ± 0.18	<0.001
LDL‐C (mmol/L)	3.03 ± 0.74	2.73 ± 0.67	2.94 ± 0.67	3.19 ± 0.74	3.26 ± 0.78	<0.001
Diabetes and prediabetes						<0.001
No	2061 (92.8)	539 (97.3)	533 (95.9)	512 (92.3)	477 (85.9)	
Yes	159 (7.2)	15 (2.7)	23 (4.1)	43 (7.7)	78 (14.1)	

Abbreviations: HDL‐C, high‐density lipoprotein; LDL‐C, low‐density lipoprotein; TG, triglyceride.

The participants were further categorized into four groups based on mean AIP levels: Q1 (<−0.3398, *n* = 554), Q2 (−0.3398 to −0.1610, *n* = 556), Q3 (−0.1610 to −0.0613, *n* = 555), and Q4 (>0.0613, *n* = 555). Participants in the highest quartile of AIP, compared with those in the lowest quartile, were more likely to be older, have a higher BMI, and be married. They also had lower levels of education, were more frequently smokers and drinkers, less physically active, and exhibited higher levels of blood glucose, TC, TG, and LDL‐C, along with lower HDL‐C concentrations.

### 3.2 Associations of the AIP with prediabetes and diabetes

**FIGURE 1:**
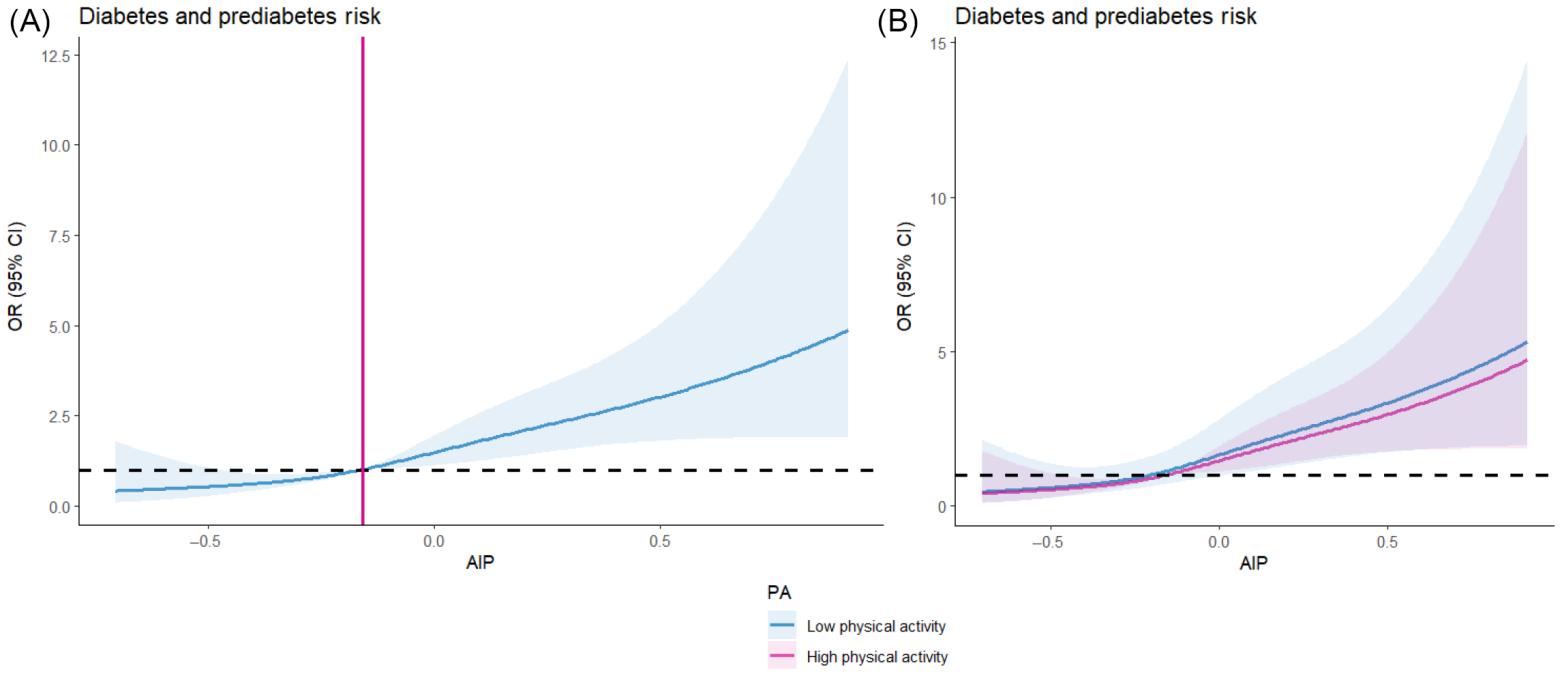
Atherogenic index of plasma (AIP) was associated with a restricted cubic spline (RCS) of prediabetes and diabetes prevalence and stratified by physical activity. CI, confidence interval; OR, odds ratio.

Table 2 shows the logistic regression results, indicating a positive association of AIP with prediabetes and diabetes. After adjusting for gender, age, BMI and TC, LDL‐C, smoking, drinking, marital status, education, the ORs (95% CI) for Q2, 3, and 4 compared with Q1 of AIP were 1.322 (0.675–2.589), 2.285 (1.219–4.284), and 3.447 (1.829–6.497) (*p* for trend <0.001), respectively. RCS analyses assessed the linear relationship between AIP, used as a continuous variable, and prediabetes and diabetes (Figure 1A). The prevalence of prediabetes and diabetes is significantly enhanced at AIP values greater than −0.16. (*p* for linearity <0.05).

**TABLE 2:** Atherogenic index of plasma (AIP) associated with prediabetes and diabetes prevalence, according to the physical activity (PA) stratification.

	Sample	OR (95% CI)		
		Model1	Model2	Model3
All	159/2220 (7.2)			
Q1		‐	‐	‐
Q2		1.551 (0.800–3.004)	1.461 (0.753–2.838)	1.322 (0.675–2.589)
Q3		3.018 (1.656–5.499)*	2.692 (1.466–4.942)**	2.285 (1.219–4.284)**
Q4		5.876 (3.336–10.350)*	4.752 (2.626–8.597)*	3.447 (1.829–6.497)*
*p* for trend		<0.001	<0.001	<0.001
Low PA	134/1810 (7.4)			
Q1		‐	‐	‐
Q2		1.336 (0.625–2.859)	1.277 (0.596–2.735)	1.203 (0.557–2.597)
Q3		3.202 (1.647–6.277)*	2.858 (1.458–5.602)**	2.555 (1.275–5.119)**
Q4		6.135 (3.274–11.495)*	5.024 (2.604–9.693)*	3.678 (1.819–7.434)*
*p* for trend		<0.001	<0.001	<0.001
Medium‐high PA	25/410 (6.1)			
Q1		‐	‐	‐
Q2		2.446 (0.616–9.710)	2.120 (0.525–8.559)	1.358 (0.311–5.935)
Q3		2.224 (0.542–9.134)	1.993 (0.472–8.414)	1.204 (0.257–5.630)
Q4		4.419 (1.158–16.869)**	3.232 (0.780–13.384)	1.925 (0.411–9.007)
*p* for trend		0.030	0.124	0.398

*Note*: Model 1, without adjustment for any covariates. Model 2, adjusted for gender and age. Model 3, adjusted for gender, age, body mass index (BMI) and total cholesterol, low‐density lipoprotein cholesterol, smoking, drinking, marital status, and education.

Abbreviations: CI, confidence interval; OR, odds ratio.

* <0.001;

** <0.05.

### 3.3 Associations of AIP with prediabetes and diabetes by physical activity

Table 2 and Figure 1B show the connection of AIP with the prevalence of prediabetes and diabetes in the PA subgroups. In the low PA population, each quartile rises in AIP was interlinked with a significant higher in the prevalence of developing prediabetes and diabetes (OR: 3.678, 95% CI: 1.819–7.434) (*p* for trend <0.001). However, this connection was not present in the medium to high PA population, and the results were not significant (OR: 1.925, 95% CI: 0.411–9.007) (*p* for trend = 0.398), this suggests that moderate to high levels of PA modify the impact of AIP on the risk of prediabetes and diabetes mellitus. Similar outcomes were noted when AIP was used as a continuous variable (Table S1). The results for RCS are shown in Figure 1B.

Further joint analysis using the low AIP medium‐high PA population as a reference found that high AIP and low PA were significantly and positively associated with prediabetes and diabetes prevalence, with progressively higher ORs. No significant association was observed in the other three groups (AIP−/PA+, OR: 0.770, 95% CI: 0.366–1.619; AIP+/PA−, OR: 1.403, 95% CI: 0.604–3.257; AIP+/PA+, OR: 2.021, 95% CI: 1.019–4.011) (Table 3). This is consistent with our previous findings that the impact on prediabetes and diabetes is more pronounced when AIP levels are higher and PA is lower.

**TABLE 3:** Joint analysis of atherogenic index of plasma (AIP) and physical activity (PA).

Variables	Sample	Model 1	Model 2	Model 3
		OR (95% CI)	OR (95% CI)	OR (95% CI)
Low AIP, medium‐high PA (AIP−/PA−)	223/2220 (10.0)	‐	‐	‐
Low AIP, low PA (AIP−/PA+)	887/2220 (40.0)	0.694 (0.332–1.452)	0.777 (0.370–1.632)	0.770 (0.366–1.619)
High AIP, medium‐high PA (AIP+/PA−)	187/2220 (8.4)	1.858 (0.814–4.239)	1.637 (0.710–3.774)	1.403 (0.604–3.257)
High AIP，low PA (AIP+/PA+)	923/2220 (41.6)	2.764 (1.420–5.377)	2.545 (1.301–4.978)	2.021 (1.019–4.011)

*Note*: Model 1, without adjustment for any covariates. Model 2, adjusted for gender and age. Model 3, adjusted for gender, age, body mass index (BMI) and total cholesterol, low‐density lipoprotein cholesterol, smoking, drinking, marital status, and education.

Abbreviations: CI, confidence interval; OR, odds ratio.

### 3.4 Sensitivity analyses and subgroup analyses

The results of the sensitivity and subgroup analyses are detailed in Tables S2–S6. Most results are consistent with the main analysis. We found a positive correlation between AIP and FBG values in linear regression (*β*: 0.608/0.224, 95% CI: 0.477–0.738). For each unit increase in AIP, the correlation was stronger in the low PA population (*β*: 0.609/0.224, 95% CI: 0.462–0.755) than in the medium‐high PA population (*β*: 0.422/0.147, 95% CI: 0.133–0.711) (Table 4). It shows that our results are robust. Additionally, in the subgroup analyses, when AIP was treated as a categorical or continuous variable, only the female population and those aged less than 45 years had results consistent with the main analysis, this indicates that there is heterogeneity in terms of sex and age.

**TABLE 4:** Sensitivity analysis of the association between continuous atherogenic index of plasma (AIP) and fasting glucose, stratified by physical activity (PA).

	Sample	Line regression β (95% CI)		
		Model 1	Model 2	Model 3
All	159/2220 (7.2)	0.819 (0.712–0.927)*	0.696 (0.579–0.813)*	0.608 (0.477–0.738)*
Low PA	134/1810 (7.4)	0.849 (0.730–0.968)*	0.712 (0.581–0.844)*	0.609 (0.462–0.755)*
Medium‐high PA	25/410 (6.1)	0.622 (0.370–0.873)*	0.509 (0.241–0.777)*	0.422 (0.133–0.711)**

*Note*: Model 1, without adjustment for any covariates. Model 2, adjusted for gender and age. Model 3, adjusted for gender, age, body mass index (BMI) and total cholesterol, low‐density lipoprotein cholesterol, smoking, drinking, marital status, and education.

Abbreviation: CI, confidence interval.

* <0.001;

** <0.05.

## 4 DISCUSSION

In this study involving 2220 young and middle‐aged participants, we found that the prevalence of prediabetes and diabetes increased with higher AIP levels. PA significantly modified this association, with adherence to moderate to high levels of PA potentially reducing the diabetes risk associated with elevated AIP levels.

There are several studies on AIP and prediabetes and diabetes, mostly focused on North America and Asia. Two studies from the United States each found that AIP is significantly correlated with IR, prediabetes, and an improved risk of diabetes in adults over 18 years old.^15^, ^25^ One of these studies examined gender differences and found that these associations were observed only in women. Our subgroup analyses showed similar results. Additionally, a cohort study in China indicated that AIP was strongly related to the risk of prediabetes in adults when AIP was ≤0.03; however, no significant association was found for AIP values above this threshold.^14^ Overall, our findings were similar to theirs, despite some heterogeneity in the subgroup analyses.

PA regulates metabolic levels and directly reduces AIP values, as confirmed by several studies. Mika Venojärvi et al.^26^ conducted a 12‐week randomized controlled trial and found that aerobic off‐road walking reduced AIP levels in middle‐aged men. Another survey showed that moderate‐intensity aerobic exercise lasting 90 min or more per week significantly decreased AIP values.^27^ However, none of these prior studies reported whether PA influences the relationship between AIP and the risk of prediabetes and diabetes in young and middle‐aged populations. Compared with other studies, our study offers a novel perspective on the association between AIP and diabetes. First, to the best of our knowledge, this is the first study to examine the relationship between AIP, PA, and prediabetes and diabetes and identify a correlation among these factors, providing novel evidence for the prevention and management of diabetes. Second, when we tested the robustness of our results through a series of sensitivity analyses (target independent variable transformations, subgroup analyses), we found stronger positive correlations in females and in participants younger than 45 years, which may be of clinical interest as the onset of diabetes occurs at younger ages. Third, our study confirms that the association between AIP, PA, and prediabetes and diabetes remains robust. More importantly, our findings highlight the importance of targeting AIP levels in clinical interventions to reduce the risk of prediabetes and diabetes. Early intervention could improve prognosis if more vigorous PA or therapeutic measures are implemented to reduce AIP at an early stage.

Our research showed that the association between AIP and prediabetes and diabetes varied by PA. We speculate that the potential causes are as follows. First, exercise can stimulate the secretion of growth hormone, promote the growth and regeneration of pancreatic β cells, and enhance their activity, thus increasing insulin secretion, which helps maintain insulin balance in the body.^28^ Improvements in β‐cell function have been reported in patients with type 2 diabetes mellitus after moderate high‐intensity interval training. Additionally, PA increases adenosine monophosphate‐activated protein kinase (AMPK) activity, and AMPK activation inactivates tre‐2/USP6, BUB2, cdc16 domain family member 1(TBC1D1), promoting the expression and activity of glucose transporter (GLUT) on cell membranes.^29^, ^30^ This helps cells uptake and utilize glucose, and the improvement in insulin sensitivity is associated with increased AMPK activity.^31^ In our study, AIP computed from TG and HDL‐C. Higher concentrations of TG affect β‐cell function and survival by increasing endoplasmic reticulum stress, causing oxidative stress, and inducing an inflammatory response, among other pathways.^32^, ^33^, ^34^ This affects normal insulin secretion and blood glucose regulation, potentially contributing to the development of diabetes. HDL‐C is engaged in cholesterol transport, and lower concentrations of HDL‐C may affect the stability of cell membranes, compromising the structure and function of insulin receptors, thus affecting insulin secretion and sensitivity.^14^, ^35^, ^36^, ^37^ These potential mechanisms may provide a pathophysiological explanation for the connections for AIP, PA, and the prevalence of prediabetes and diabetes.

The strengths of this study include being the first to examine the association between AIP, PA, and prediabetes and diabetes in a young and middle‐aged population, and reporting the threshold value of AIP. When the AIP of young and middle‐aged people is >−0.16, the prevalence of prediabetes and diabetes increases, highlighting the importance of early detection, diagnosis, and treatment. Additionally, we conducted two sensitivity analyses to verify the relationship from different perspectives. This study also have some limitations. First, our study is cross‐sectional, so it cannot infer cause and effect among the three factors. However, follow‐up investigations of this cohort are ongoing, allowing future use of longitudinal data to further investigate these associations. Second, our PA and certain assessments were based on patient self‐reports, potentially introducing biases. Finally, although some relevant potential confounders were adjusted for in our study, we were unable to account for all potential confounders due to data limitations, including biochemical markers such as serum creatinine, aspartate aminotransferase, alanine aminotransferase, and family history of diabetes.^14^ There is evidence that these factors are associated with prediabetes risk. Therefore, future studies should aim to include a broader range of variables to minimize the impact of confounding factors.

## 5 CONCLUSIONS

In the present study, we found that increasing PA levels in young and middle‐aged populations attenuated the unfavorable association of AIP with prediabetes and diabetes. Additionally, greater AIP was significantly linked to prediabetes and diabetes prevalence in young and middle‐aged patients with AIP > −0.16. These findings suggest measures for diabetes prevention and treatment. However, further investigations are required to confirm these causal relationships and potential mechanisms.

## AUTHOR CONTRIBUTIONS

Shenglan Yang contributed to study design, data collection, statistical analysis, interpretation of the results, and manuscript writing; Xinyu Gou, Hui Dong, Limei Chen, Yiyan Wang contributed to data collection and analysis; Jing Wu contributed to study design and interpretation, critical revision of the manuscript, and funding.

## FUNDING INFORMATION

The study was supported by Shanghai Natural Science Foundation (23ZR1463600).

## CONFLICT OF INTEREST STATEMENT

The authors declare no conflicts of interest.

## INFORMED CONSENT STATEMENT

Every participant willingly engaged in the study and submitted a written informed consent for both their participation.

## Supporting information


**Table S1.** Logistic regression of AIP as a continuous variable with prediabetes and diabetes mellitus.
**Table S2.** AIP is a linear regression of categorical variables against glucose.
**Table S3.** AIP as logistic regression of categorical variables with prediabetes and diabetes, with subgroup analyses by gender.
**Table S4.** AIP as logistic regression of categorical variables with prediabetes and diabetes, with subgroup analyses by age.
**Table S5.** AIP as logistic regression of continuous variables with prediabetes and diabetes, with subgroup analyses by gender.
**Table S6.** AIP as logistic regression of continuous variables with prediabetes and diabetes, with subgroup analyses by age.

## Data Availability

Data will be available upon request.
