# Supplementary material for: Physical activity modifies the association between atherogenic index of plasma and prediabetes and diabetes: A cross‐sectional analysis
Source: J Diabetes. 2024 Oct 13;16(10):e70006. doi: 10.1111/1753-0407.70006 (PMC11471440; doi:10.1111/1753-0407.70006)
Supplement: Supplementary file 1 — Table S1. Logistic regression of AIP as a continuous variable with prediabetes and diabetes mellitus. Table S2. AIP is a linear regression of categorical variables against glucose. Table S3. AIP as logistic regression of categorical variables with prediabetes and diabetes, with subgroup analyses by gender. Table S4. AIP as logistic regression of categorical variables with prediabetes and diabetes, with subgroup analyses by age. Table S5. AIP as logistic regression of continuous variables with prediabetes and diabetes, with subgroup analyses by gender. Table S6. AIP as logistic regression of continuous variables with prediabetes and diabetes, with subgroup analyses by age. [file JDB-16-e70006-s001.docx]

Table S1 Logistic regression of AIP as a continuous variable with prediabetes and diabetes mellitus

|  | Sample |  | OR (95%CI) |  |
| --- | --- | --- | --- | --- |
|  |  | Model1 | Model2 | Model3 |
| all | 159/2220(7.2) | 7.683(4.834,12.212)** | 6.353(3.805,10.606)** | 5.496(2.912,10.374)** |
| low physical activity | 20/327(6.1) | 8.045(4.874,13.278)** | 6.715(3.846,11.724)** | 5.410(2.698,10.849)** |
| medium-high physical activity | 139/1893(7.3) | 5.446(1.534,19.341)* | 4.271(1.029,17.736)* | 4.057(0.690,23.840) |

*<0.05, **<0.001

Model1, without adjustment for any covariates

Model2, adjust gender, age

Model3, adjust gender, age, BMI and total cholesterol, low-density lipoprotein cholesterol, smoking, drinking, marry state, Education.

Table S2 AIP is a linear regression of categorical variables against glucose

|  | Sample | Line Regression β (95%CI) | | |
| --- | --- | --- | --- | --- |
|  |  | Model1 | Model2 | Model3 |
| all | 159/2220(7.2) |  |  |  |
| Q1 |  | - | - | - |
| Q2 |  | 0.095(-0.001,0.190) | 0.069(-0.025,0.164) | 0.049(-0.046,0.144) |
| Q3 |  | 0.244(0.149,0.340)** | 0.187(0.091,0.283)** | 0.150(0.049,0.250)* |
| Q4 |  | 0.566(0.470,0.661)** | 0.446(0.344,0.547)** | 0.354(0.244,0.464)** |
| P for trend |  | <0.001 | <0.001 | <0.001 |
| low PA | 134/1810(7.4) |  |  |  |
| Q1 |  | - | - | - |
| Q2 |  | 0.089(-0.020,0.198) | 0.066(-0.042,0.174) | 0.061(-0.047,0.170) |
| Q3 |  | 0.252(0.143,0.361)** | 0.185(0.075,0.294)** | 0.170(0.057,0.284)* |
| Q4 |  | 0.577(0.470,0.685)** | 0.443(0.329,0.558)** | 0.351(0.227,0.476)** |
| P for trend |  | <0.001 | <0.001 | <0.001 |
| medium-high PA | 25/410(6.1) |  |  |  |
| Q1 |  | - | - | - |
| Q2 |  | 0.116(-0.077,0.308) | 0.081(-0.111,0.272) | -0.009(-0.204,0.186) |
| Q3 |  | 0.206(0.010,0.402)* | 0.173(-0.023,0.368) | 0.038(-0.170,0.246) |
| Q4 |  | 0.481(0.010,0.402)** | 0.377(0.158,0.597)** | 0.258(0.026,0.490)* |
| P for trend |  | <0.001 | <0.001 | 0.026 |

*<0.05, **<0.001

Model1, without adjustment for any covariates

Model2, adjust gender, age

Model3, adjust gender, age, BMI and total cholesterol, low-density lipoprotein cholesterol, smoking, drinking, marry state, Education.

Table S3 AIP as logistic regression of categorical variables with prediabetes and diabetes, with subgroup analyses by gender

|  | Sample | OR (95%CI) | | |
| --- | --- | --- | --- | --- |
|  |  | Model1 | Model2 | Model3 |
| Female |  |  |  |  |
| all | 98/1710(5.7) |  |  |  |
| Q1 |  | - | - | - |
| Q2 |  | 1.399(0.688,2.845) | 1.380(0.678,2.808) | 1.222(0.593,2.516) |
| Q3 |  | 2.802(1.466,5.356)* | 2.757(1.441,5.275)* | 2.201(1.114,4.349)* |
| Q4 |  | 5.123(2.713,9.671) | 4.983(2.634,9.424)** | 3.355(1.672,6.734)** |
| P for trend |  | <0.001 | <0.001 | <0.001 |
| low physical activity | 85/1429(5.9) |  |  |  |
| Q1 |  | - | - | - |
| Q2 |  | 1.355(0.608,3.021) | 1.341(0.601,2.991) | 1.202(0.532,2.713) |
| Q3 |  | 3.150(1.539,6.447)* | 3.091(1.508,6.333)* | 2.518(1.190,5.328)* |
| Q4 |  | 5.518(2.736,11.128)** | 5.379(2.661,10.872)** | 3.664(1.706,7.870)** |
| P for trend |  | <0.001 | <0.001 | <0.001 |
| medium-high physical activity | 13/281(4.6) |  |  |  |
| Q1 |  | - | - | - |
| Q2 |  | 1.607(0.349,7.397) | 1.551(0.335,7.170) | 1.143(0.231,5.650) |
| Q3 |  | 1.424(0.279,7.276) | 1.462(0.285,7.488) | 0.974(0.162,5.859) |
| Q4 |  | 3.133(0.600,16.352) | 3.033(0.579,15.895) | 1.619(0.244,10.742) |
| P for trend |  | 0.220 | 0.224 | 0.670 |
| Male |  |  |  |  |
| all | 61/510(12.0) |  |  |  |
| Q1 |  | -- | - | - |
| Q2 |  | 2.426(0.272,21.615) | 2.034(0.223,18.587) | 1.909(0.206,17.728) |
| Q3 |  | 3.432(0.433,27.193) | 2.702(0.334,21.874) | 2.570(0.310,21.294) |
| Q4 |  | 6.215(0.827,46.692) | 4.851(0.633,37.163) | 4.039(0.505,32.332) |
| P for trend |  | 0.003 | 0.008 | 0.045 |
| low physical activity | 49/381(12.9) |  |  |  |
| Q1 |  | - | - | - |
| Q2 |  | 0.857(0.073,10.064) | 0.688(0.056,8.400) | 0.664(0.053,8.246) |
| Q3 |  | 1.935(0.233,16.071) | 1.423(0.165,12.286) | 1.342(0.149,12.092) |
| Q4 |  | 3.620(0.468,27.987) | 2.637(0.329,21.145) | 2.081(0.239,18.127) |
| P for trend |  | 0.008 | 0.018 | 0.103 |
| medium-high physical activity^†^ | 12/129(9.3) |  |  |  |

*<0.05, **<0.001

^†^No data were allocated to this group; hence, no results are reported.

Model1, without adjustment for any covariates

Model2, adjust gender, age

Model3, adjust gender, age, BMI and total cholesterol, low-density lipoprotein cholesterol, smoking, drinking, marry state, Education.

Table S4 AIP as logistic regression of categorical variables with prediabetes and diabetes, with subgroup analyses by age

|  | Sample | OR (95%CI) | | |
| --- | --- | --- | --- | --- |
|  |  | Model1 | Model2 | Model3 |
| ≥45y |  |  |  |  |
| all | 56/509(11.0) |  |  |  |
| Q1 |  | - | - | - |
| Q2 |  | 2.086(0.539,8.073) | 1.827(0.468,7.129) | 1.806(0.455,7.171) |
| Q3 |  | 3.593(0.995,12.972) | 2.679(0.725,9.894) | 2.702(0.715,10.205) |
| Q4 |  | 8.997(2.672,30.289)** | 5.325(1.502,18.887)* | 4.868(1.305,18.151)* |
| P for trend |  | <0.001 | <0.001 | 0.04 |
| low physical activity | 45/405(11.1) |  |  |  |
| Q1 |  | - | - | - |
| Q2 |  | 1.246(0.288,5.385) | 1.089(0.249,4.751) | 1.153(0.258,5.151) |
| Q3 |  | 2.770(0.745,10.298) | 2.091(0.546,8.002) | 2.363(0.603,9.263) |
| Q4 |  | 6.153(1.793,21.119)* | 3.634(0.980,13.468) | 3.409(0.860,13.512) |
| P for trend |  | <0.001 | 0.007 | 0.027 |
| medium-high physical activity^†^ | 11/104(10.6) |  |  |  |
| <45y |  |  |  |  |
| all | 103/1608(6.0) |  |  |  |
| Q1 |  | - | - | - |
| Q2 |  | 1.351(0.625,2.920) | 1.362(0.630,2.945) | 1.216(0.558,2.651) |
| Q3 |  | 2.797(1.413,5.539)* | 2.861(1.439,5.688)* | 2.291(1.117,4.699)* |
| Q4 |  | 4.680(2.443,8.969)** | 4.929(2.511,9.673)** | 3.188(1.526,6.660)* |
| P for trend |  | <0.001 | <0.001 | <0.001 |
| low physical activity | 89/1405(6.3) |  |  |  |
| Q1 |  | - | - | - |
| Q2 |  | 1.314(0.538,3.210) | 1.319(0.540,3.223) | 1.187(0.481,2.927) |
| Q3 |  | 3.241(1.496,7.020)* | 3.290(1.512,7.159)* | 2.686(1.196,6.034)* |
| Q4 |  | 5.661(2.716,11.800)** | 5.856(2.742,12.508)** | 3.796(1.664,8.659)* |
| P for trend |  | <0.001 | <0.001 | <0.001 |
| medium-high physical activity | 14/306(4.6) |  |  |  |
| Q1 |  | - | - | - |
| Q2 |  | 1.476(0.320,6.809) | 1.511(0.325,7.031) | 1.145(0.229,5.717) |
| Q3 |  | 1.476(0.320,6.809) | 1.521(0.325,7.115) | 1.118(0.204,6.132) |
| Q4 |  | 1.407(0.274,7.214) | 1.529(0.271,8.617) | 0.859(0.120,6.154) |
| P for trend |  | 0.709 | 0.650 | 0.849 |

*<0.05, **<0.001

^†^No data were allocated to this group; hence, no results are reported.

Model1, without adjustment for any covariates

Model2, adjust gender, age

Model3, adjust gender, age, BMI and total cholesterol, low-density lipoprotein cholesterol, smoking, drinking, marry state, Education.

Table S5 AIP as logistic regression of continuous variables with prediabetes and diabetes, with subgroup analyses by gender

|  | Sample | OR (95%CI) | | |
| --- | --- | --- | --- | --- |
|  |  | Model1 | Model2 | Model3 |
| Female |  |  |  |  |
| all | 98/1612(5.7) | 9.179(4.697,17.936)** | 8.907(4.548,17.443)** | 5.924(2.717,12.915)** |
| low PA | 85/1429(5.9) | 9.545(4.709,19.347)** | 9.277(4.562,18.866)** | 6.209(2.715,14.202)** |
| medium-high PA | 13/281(4.6) | 5.417(0.584,50.222) | 5.422(0.585,50.249) | 2.591(0.184,36.438) |
| Male |  |  |  |  |
| all | 61/510(12.0) | 4.627(2.165,9.891)** | 4.208(1.943,9.110)** | 5.379(1.699,17.032)* |
| low PA | 49/381(12.9) | 4.375(2.004,11.184)** | 4.177(1.755,9.942)* | 4.539(1.134,18.167) |
| medium-high PA | 12/129(9.3) | 3.639(0.616,21.484) | 3.877(0.583,25.777) | 8.198(0.551,121,889) |

*<0.05, **<0.001

Model1, without adjustment for any covariates

Model2, adjust gender, age

Model3, adjust gender, age, BMI and total cholesterol, low-density lipoprotein cholesterol, smoking, drinking, marry state, education.

Table S6 AIP as logistic regression of continuous variables with prediabetes and diabetes, with subgroup analyses by age

|  | Sample | OR (95%CI) | | |
| --- | --- | --- | --- | --- |
|  |  | Model1 | Model2 | Model3 |
| age≥45y |  |  |  |  |
| all | 56/509(11.0) | 11.514(4.995,26.539)** | 6.796(2.708,17.054)** | 9.446(2.887,30.905)** |
| low PA | 45/405(11.1) | 11.288(4.454,28.607)** | 7.028(2.486,19.870)** | 7.249(1.928,27.255)* |
| medium-high PA | 11/104(10.6) | 15.140(1.924,119.107)* | 7.005(0.809,60.614) | 227.522(2.085,24833.069)* |
| age＜45y |  |  |  |  |
| all | 103/1711(6.0) | 5.879(3.340,10.349)** | 6.605(3,565,12.240)** | 4.674(2.159,10.119)** |
| low PA | 89/1405(6.3) | 6.469(3.551,11.786)** | 7.062(3.662,13.620)** | 5.298(2.296,12.227)** |
| medium-high PA | 14/306(4.6) | 2.091(0.333,13.142) | 2.531(0.341,18.761) | 1.395(0.132,14.763) |

*<0.05, **<0.001

Model1, without adjustment for any covariates

Model2, adjust gender, age

Model3, adjust gender, age, BMI and total cholesterol, low-density lipoprotein cholesterol, smoking, drinking, marry state, education.
